# Adaptation of Sonication-Assisted Matrix Solid Phase Dispersion of Tissues for the Subsequent Extraction of Polycyclic Aromatic Hydrocarbons from Gulf Menhaden (*Brevoortia patronus*)

**DOI:** 10.1155/2014/925684

**Published:** 2014-03-09

**Authors:** Gregory M. Olson, Buffy M. Meyer, Ralph J. Portier

**Affiliations:** Department of Environmental Sciences, 1273 Energy, Coast & Environment Building, Louisiana State University, Baton Rouge, LA 70803, USA

## Abstract

A new adaptation based on matrix solid phase dispersion of tissue for the subsequent isolation of polycyclic aromatic hydrocarbons was developed and used for extractions of Gulf menhaden caught during the summer of 2011. Many Matrix Solid Phase Dispersion (MSPD) methods require specific cartridges and other clean-up materials in order to achieve proper extraction. For this study, the tissues were lyophilized prior to applying the adapted MSPD method allowing for a much more complete homogenization with the C18 silica. The tissue was spiked with phenanthrene d_10_ as a surrogate as a measure of PAH recovery prior to the lyophilisation process to determine if any target compounds were lost and prior to sonication as per the finalized adaptation procedure to determine method efficiency. This technique used C18 silica in a 1 : 1 ratio as the primary homogenizing material for the menhaden tissue matrix and was eluted with dichloromethane (DCM) until visibly clear. The overall study mean recovery was 88% ± 5% with method detection limits between 0.4 ng/g and 4.4 ng/g tissue dry weight. This adapted protocol has been used exclusively on the analysis of high lipid content fish stocks affected by dispersed and weathered oil from the BP Horizon incident.

## 1. Introduction

The release of large quantities of crude oil into the Gulf of Mexico during the 2010 BP Deepwater Horizon incident has raised concerns based on contamination of marine organisms with constituents of weathered crude oil. One major group of compounds found in crude oil that is of major concern is the Polycyclic Aromatic Hydrocarbons (PAHs) [1]. This group of compounds can be characterized by multiple conjoined ring structures with naphthalene and its alkylated forms being the smallest (molecular mass of 128.17 g/mol) [2, 3]. The higher molar mass of theses PAHs results in less volatilization, which in turn allows those compounds to remain in nature far longer than other constituents of oil [4]. This leads to the possibility of bioaccumulation within the adipose fraction of marine organisms and possible biomagnification within the trophic structure of the Gulf of Mexico [5].

PAHs are considered compounds of concern according to the US Environmental Protection Agency due to their ability to accumulate within adipose tissue [6]. Several PAHs are considered mutagenic as well as carcinogenic, making their possible presence in a commercially important fish such as menhaden a major concern [6]. The Gulf menhaden (*Brevoortia patronus*) was identified as the largest commercial harvest from the Gulf of Mexico and was selected as the principle organism to study [7]. The menhaden were collected due to the amount of fats and oils that can be extracted from them and refined for consumer use, which is important because of the lipophilic nature of PAHs [8, 9]. This fish also plays a key role in the trophic structure of the Gulf of Mexico acting as the main forage fish for many species of fish, dolphins, and waterfowl [8]. This obligate filter feeder has two very important factors contributing to its selection as a sentinel species: (1) the menhaden are in contact with surface and subsurface oil through dermal exposure and direct ingestion; and (2) due to sheer fish stock volumes and trophic predation, the menhaden are the main link between producers and secondary consumers [7–9].

The matrix solid phase dispersion (MSPD) method is an extraction method identified by the total disruption of the sample through the use of an appropriate bonded phase or other solid support material such as octadecylsilyl (ODS-) derivatized silica (C18 silica) being ground with the sample. This creates a new phase consisting of the sample and bonded phase material and allows for distinctive sample fractionation [10, 11]. For this experiment, a lipophilic bonding phase of C18 silica was used; however, the use of C-8 silica is considered a possible alternative [10]. The form of MSPD extraction used in the study can be described as vacuum assisted because of the vacuum applied to the apparatus after gravity filtration has completed. Generally, the eluent collected from this process is sufficiently “clean” for direct injection into analytical instruments. However, additional clean-up measures can be conducted such as cocolumn clean-up where the addition of other support materials are added to the bottom of the container [10].

The goal of this study is to determine if the outlined adaptations to MSPD extraction techniques will result in valid and quantifiable data for use in monitoring waters impacted by oil spill events.

## 2. Materials and Methods

### 2.1. Solvents, Reagents, and Chemicals

Pesticide reagent grade solvents were used in all standard preparations, sample analysis, and rinsing procedures. The dichloromethane (DCM) and hexane (Mallinckrodt Chemicals) and RediSep C18 silica (40–60 *μ*m, Teledyne Isco) were used for tissue extraction. Sodium sulfate (anhydrous, 10–60 mesh, Fisher Scientific) was used for final sample preparation.

### 2.2. Gulf Menhaden

The menhaden were sampled at locations around Grand Isle, Louisiana (GI), and Vermilion Bay, Louisiana (VB). The samples were harvested using a standard 5-panel gill net. This net was approximately 200 m in length with 5 distinct plastic mesh panels. The menhaden were separated by length, bagged in plastic freezer bags, and placed on ice until frozen to −4°C in a laboratory setting. Prespill (July 2009) menhaden tissue control samples were created from processed menhaden donated by a prominent menhaden processing company located in Louisiana. Fish oil and meal were combined in a ratio consistent with oil yields reported in this study for size appropriate tissue concentrations.

### 2.3. Calibration Standards

A commercially prepared crude oil analysis standard (Oil Analysis Standard, Part # 90311, Absolute Standards) was used to prepare the five-point calibration standards (0.5 ppm, 1.0 ppm, 5.0 ppm, 10.0 ppm, and 25.0 ppm) using the internal standard method for determining concentrations. Calibration standard solutions were stored in amber vials with PTFE-lined caps. The calibration standards were checked frequently for signs of degradation or evaporation and replaced if indicated in laboratory quality control checks. A continuing calibration standard (one point of the initial five-point calibration standard) was analyzed in each batch of extracted tissue samples or during each 12-hour period during which analyses were performed. Acceptance criterion for the continuing calibration standard was ±20% of the mean relative response factor calculated from the initial five-point curve. If the acceptance criterion was not met, all analyses were discontinued until the instrument was realigned to meet optimal operations criteria. With instrument maintenance or troubleshooting, a new five-point calibration curve was generated as per good laboratory practices.

The calibration standard is used to ensure consistency in the instrument response when identifying compounds. Each time the instrument source is adjusted or the column is clipped or altogether changed, all five calibration concentrations are analyzed and used as a means to determine instrument quality. The continuing calibration that accompanies all sample batches is one concentration of the five initial calibration standards and is run to ensure accurate measurement of the detector (EPA SW-846 method 8000 B) [12]. The mean response factor for each analyte is also calculated during the process of the initial five-point calibration and is used to determine analyte concentration which can be seen in the equation found in subsection Internal Standard Solution.

### 2.4. Internal Standard Solutions

Internal standards were naphthalene-d_8_ (Part # Z-014J-4), acenaphthene-d_10_ (Part # Z-014J-1), chrysene-d_12_ (Part # Z-014J-2), and perylene-d_12_ (Part # Z-014J-5) all purchased from AccuStandard Inc., New Haven, CT and stored individually until combined to make 4 mL of the internal standard injecting solution. Each internal standard is used to determine the concentrations of analytes with similar molecular weights. This is done by spiking each GC vial with 10 *μ*L of the prepared internal standard solution (10 *μ*L in 1 mL of sample) and then standardizing each target response to the known concentrations of the four standards. Once this is complete, the analyte target response can then be converted to a concentration using the formula below:

(1) Analyte  Concentration=((Target  Response)  ×(Internal  Standard  Concentration)  ×(Final  Volume)×(Dilution  Factor))    ×((Response  of  Internal  Standard)     ×(Analyte  Mean  Response  Factor)     ×(Volume  Injected)×(Dry  Mass))−1.

### 2.5. Reference Oil Standard

The usual laboratory reference oil established by USEPA has been Alaska North Slope Crude Oil (ANSCO); however, the reference oil standard used for these analyses was Macondo 252 (MC 252) collected directly from the riser of the Deepwater Horizon oil rig. Reference oil standards were prepared by extracting 1 gram of pure oil in 40 mL of solvent (or equivalent ratio of 1 g : 40 mL, e.g., 0.50 g : 20 mL). The laboratory reference oil was analyzed in each sample batch as an additional QA/QC sample, that is, a laboratory control sample.

### 2.6. Surrogate Spiking Standards

The surrogate spiking standards were 5-alpha androstane (Part # GRH-IS-10X, AccuStandard) and 10 mg of phenanthrene-d_10_ neat (Part # 364622, Sigma-Aldrich, St. Louis, MO) combined with 500 mL of DCM to make the needed concentration. The extraction efficiency for each sample was based on percent recovery of surrogate standard with an acceptable percent recovery range of 70–120% [13].

### 2.7. Preparation of the Sample Extracts

The frozen menhaden were weighed and their fork lengths were taken. Triplicate composite samples of menhaden with fork lengths of 16 cm or less (small) were selected from each field location and then chopped into small cubes approximately 12 mm × 24 mm × 24 mm. These pieces were then placed into precleaned/solvent rinsed 200 mL beakers. The cubed tissue was then compressed into the base of the beaker with a clean glass pestle, placed in a −86°C freezer and allowed to freeze. The surrogate spiking solution was added prior to freeze-drying in 7 individuals to determine if lyophilization affected recovery. Frozen samples were then freeze-dried for 24 hours (VirTis, Model Freezemobile 6). This process was repeated for menhaden with fork lengths greater than 16 cm (large) from each field location. Dried samples were placed in a dessicator prior to solvent extraction. It is important to note that this step is performed with no less than 18 samples. Batch lyophilisation is crucial in reducing overall extraction time.

**Figure 1:**
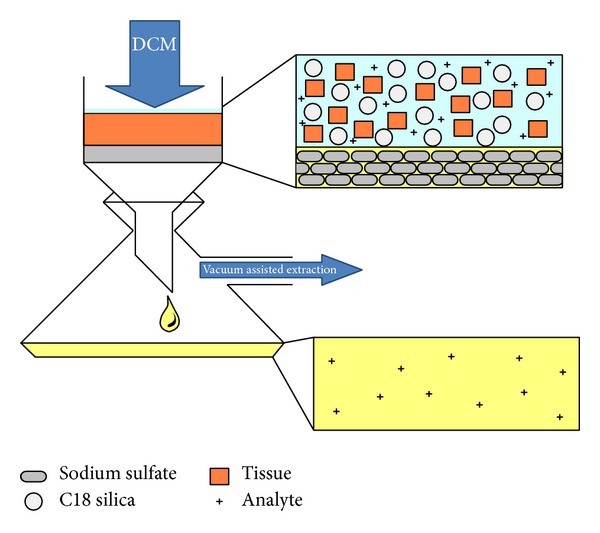
Extraction apparatus.

Desiccated fish tissue was pulverized to a fine powder and a 10 g subsample (as little as 2.5 g can be used) was removed and amended at a 1 : 1 ratio with C18 silica. Sodium sulfate in excess of 2–5 g was added and mixed in with a spatula to bind up excess moisture. Samples were then spiked with 1 mL of the surrogate spiking solution. Samples were then filled with 50 mL DCM and sonicated (Branson 2210) for 30 minutes. After the sonication process, each sample was gravity filtered through a Fisherbrand filter (09-801-G, 24 cm diameter) covered with a 10 g layer of sodium sulfate. The container used to lyophilize and sonicate the sample was rinsed three times with DCM into the homogenized sample to ensure complete transfer of all materials. The funnel (Corning, 6120-6) was attached to a side-arm flask (Corning, 5340-250) affixed to a vacuum manifold. After gravity filtration stopped, a slight vacuum (vacuum-assisted solvent extraction) was applied to finish the removal of all DCM. The resulting eluent was then moved to a flat bottom Florence flask (Corning, 4060-250), using the triple DCM rinsing technique, and rotary evaporated (Rotavap Buchi Laboratory Equipment) until all excess DCM was removed.  Figure 1 illustrates the general apparatus used for this study. The residual material in the flat-bottom Florence flask was then reconstituted in hexane and transferred to a solvent rinsed glass graduated cylinder. An appropriate amount of hexane was then used to dilute the resulting material to a whole number volume in mL (this amount is not set, enough hexane is used to dilute the sample to sufficient clarity deemed by the GC/MS operator, but was usually between 15–25 mL final volume). The solution was aspirated and homogenized with a Pasture pipette to sufficiently mix the sample. A 10 mL aliquot was collected from the graduated cylinder in a volatile organic analysis (VOA) vial for long-term storage. In the case of the eluent collected from menhaden, the only secondary clean-up method employed was a settling period after the extraction process. This allowed any material large enough to pass through the filter time to precipitate out of solution. Multiple 1 mL aliquots were collected for GC/MS analysis. Samples were placed in appropriate amber autosampler vials, spiked with 10 *μ*L of the prepared internal standard, and capped and placed in refrigeration prior to GC/MS analysis.

### 2.8. Preparation of Menhaden Controls

Control menhaden facsimile tissue was formulated using meal and oil collected during June 2009 from a commercial source. Determining the appropriate oil/meal ratio for both “small” menhaden as well as “large” menhaden allowed for the creation of these facsimile controls. Datasets were generated using a Soxhlet extraction method. 10 grams of homogenized tissue were extracted using DCM for 12–18 hours. Final material was evaporated to completion and the mass of the extracted “raw” oil was recorded. Once the oil/meal ratios for each size category were determined, control facsimiles were generated. Using the calculated means of “small” (0.13 g menhaden oil/g dry tissue) and “large” (0.39 g menhaden oil/g dry tissue) menhaden oil/meal ratios, controls were created in a 150 mL beaker. The controls were subjected to the extraction procedures as outlined above.

### 2.9. Preparation of Method Detection Limits Analysis

Method Detection Limit (MDL) procedure, 0.1 mL of oil analysis calibration standard at 25 ppm, was spiked into 3 g of prepared menhaden tissue controls created as described in Section 2.8. This was repeated six more times for a total of seven replicates. One milliliter (1 mL) of surrogate standard at 20 ppm was added to each of the seven replicates prior to extraction. The samples were then extracted using the previously described adapted MSPD technique and quantified using ChemStation E.02.01.1177.

## 3. Analytical Apparatus

### 3.1. Gas Chromatograph

All GC/MS analyses used an Agilent 5890 GC system configured with a 5% diphenyl/95% dimethyl polysiloxane high resolution capillary column (30 meter, 0.25 mm ID, 0.25 micron film) directly interfaced to an Agilent 5972 mass selective detector system. An Agilent 6890 series Autoinjector was used for sample introduction into the GC/MS system. The GC flow rates were optimized to provide a required degree of separation, particularly n-C_17_ and pristane (baseline resolved), and n-C_18_ and phytane (baseline resolved). The injection (split) temperature was set at 250°C and only high temperature; a low thermal-bleed septum was used in the GC inlet. The GC was operated in temperature program mode with an initial column temperature of 60°C for 3 minutes, increased to 280°C at a rate of 5°C/minute and finally held for 3 minutes. The oven was then heated from 280°C–300°C at a rate of 1.5°C/min and held at 300°C for two minutes. Injection volume was set to 1 *μ*L. Total runtime was 65.33 minutes per sample. The interface to the MS was maintained at 280°C. Ultra High Purity (UHP) helium was the carry gas for the GC/MS system with a flow rate of 1 mL per minute.

### 3.2. Mass Spectrometer

The MS was operated in Selective Ion Monitoring (SIM) mode to maximize the detection of several trace target constituents unique to crude oil. The ionization was achieved with electron impact at 70 eV. Selected ions for each acquisition window were scanned at a rate greater >1.5 scans/sec with a dwell time of 60 milliseconds. At the start of each analysis period or every twelve hours, the MS was tuned to PFTBA, an internal instrument standard. Laboratory reference standards such as reference oil and a continuing calibration standard were also analyzed prior to the analysis of tissue/oil sample extracts. This standard operating procedure ensured quality assurance/quality control of the instrument conditions prior to sample analysis.

### 3.3. Data Analysis

The analytical method used in the study utilizes MSD ChemStation E.02.01.1177 and identifies 71 key constituents of crude oil with 43 components classified as aromatic (Table 1). This method relies on both NIST and Wiley MS databases to identify selected PAHs in the sample matrix. The significant extraction portion of this study focused on the total aromatic concentrations found within each menhaden sampling group. Samples were individually integrated and compared to the known peaks of the 71 key constituents used to identify crude oil. From the resulting integrations and retention times, the analyte concentrations in ng/g of dry wt. tissue for whole menhaden were calculated.

Limits of detection (MDL) were calculated from the GC/MS-SIM analysis of the oil analysis calibration standard along with the tissue controls created in Section 2.9 to determine that limits of quantitation were estimated from the oil analysis calibration standard at a concentration of 10 ppb. The analysis of the 10 ppb oil analysis calibration standard resulted in detection of 10 pg peaks with signal to noise ratios above 5. Therefore, assuming a 10 gram sample size and injection of 1 *μ*L out of a total extract volume of 1000 *μ*L, this translates to a detection limit of 1 ppb for the target analytes with a specific range of 0.4–4.4 ppb. The limits of quantitation (LOQ) are then derived by multiplying an approximate value of 5 ppb by a factor of 5, resulting in a LOQ of 25 ppb for all analytes.

## 4. Results and Discussion

### 4.1. Method Evaluation Using Phenanthrene d_10_ Recovery

The spiking surrogate solution containing phenanthrene d_10_ was administered at two different times of the study in order to show method validity. The samples spiked prior to lyophilizing had a mean recovery of 87% of the phenanthrene d_10_. The samples spiked after the subsample was taken had a mean recovery of 89%. The standard deviation of the samples spiked prior to the lyophilizing process was 8%, and those spiked after the lyophilizing process had a standard deviation of 4%. There was no significant loss in phenanthrene d_10_ recovery in the lyophilizing process. The adaptation of a sonication-assisted MSPD extraction yielded recoveries greater than 90%. Overall study summary statistics were (1) a recovery mean of 88%, ±5% and (2) a range from 75%–96% with a total sample size of *N* = 36 (Table 2). The lowest recorded recovery among the samples spiked after the lyophilizing process was 80%, with the lowest recorded recovery among the samples spiked before the lyophilizing process being 75%. Highest recorded recovery among the samples spiked after the lyophilizing process was 96% with the highest recorded recovery among samples spiked before the lyophilizing process being 93%.

**Figure 2:**
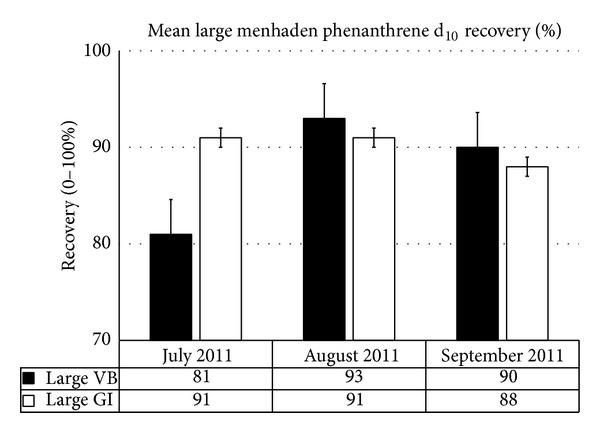
Mean “large” menhaden phenanthrene d_10_ recoveries based on site and month of harvest, Summer 2011 (recoveries by month were not significantly different *α* = 0.05).

**Figure 3:**
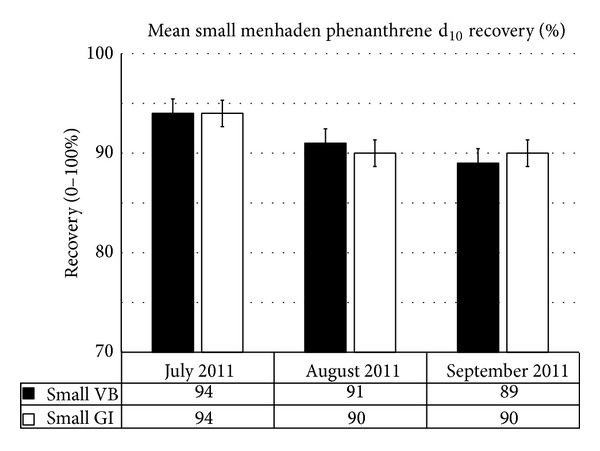
Mean “small” menhaden phenanthrene d_10_ recoveries based on site and month, Summer 2011 (recoveries by month were not significantly different *α* = 0.05).

**Figure 4:**
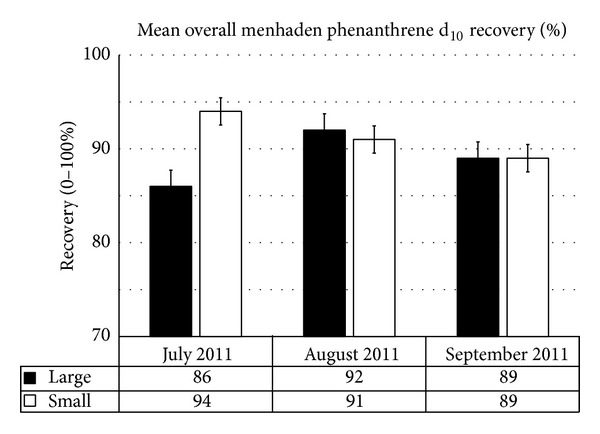
Overall mean phenanthrene d_10_ recoveries based on size and month, Summer 2011 (recoveries by month were not significantly different *α* = 0.05).

Size as a controlling factor for PAH recoveries indicated minimal variation which can be linked to life cycle. Monthly variations were also not significant for phenanthrene d_10_ recoveries. The only month with a noticeable difference in recoveries versus size and location was July 2011; however, this difference was not significant between Vermilion Bay (VB) and Grand Isle (GI) (Figure 2). Similar responses were seen for small menhaden from both VB and GI (Figure 3) and all fish harvested independent of sample site (Figure 4). The disparity noted in July, between location (Figures 2 and 3) as well as size (Figure 4), indicated that the difference in recoveries stem from human error. These inefficiencies still resulted in an 81% recovery when comparing phenanthrene d_10_ by site and an 86% recovery when comparing phenanthrene d_10_ by size, that is, well within an acceptable method range of 70%–120%. Statistical analysis of the recoveries using a one-way analysis of variance with an *α* of 0.05 showed that all sample means including prelyophilisation, postlyophilisation, and control (Table 2) demonstrated no significant difference (*P*-value = 0.57).

**Figure 5:**
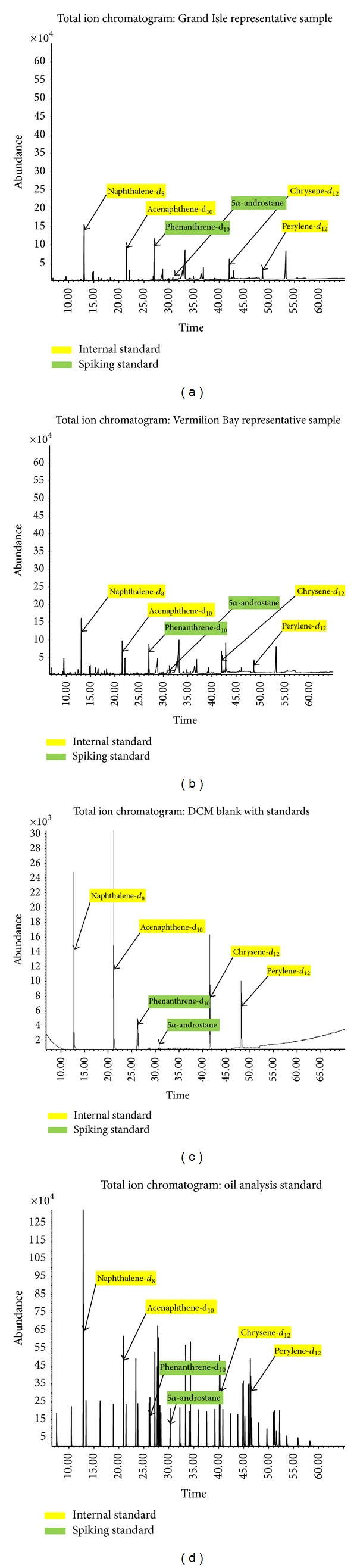
Total ion chromatograms of (a) representative Grand Isle sample, (b) representative Vermilion Bay sample, (c) method blank, and (d) oil analysis calibration standard @ 5.0 ppm.

Total ion chromatograms (TIC) are shown of representative samples to indicate separation on column as well as general relative abundance versus acquisition method time. Figures 5(a) and 5(b) are labelled to identify internal and spiking standards as within the selected sample matrix. Method blanks were analyzed throughout each batch and were all free of contamination as can be seen in Figure 5(c). The continuing calibration standards were all within 20% RSD (relative standard deviation) of the 5-point calibration mean showing consistency and precision: a representative TIC can be seen in Figure 5(d).

## 5. Conclusion

The adaptations of lyophilisation and sonication made to the gravity and then vacuum assisted MSPD extraction method improved minimum recovery by approximately 18%. The overall standard deviation of ±5% from an average recovery of 88% demonstrated minimal variation in individual sample recovery and overall recoveries were consistent. Extractions used as little as 2.5 g of sample with minimal amounts (<50 mL) of DCM needed to elute fish tissue to completion. Minimal amounts of hexane were needed to reconstitute the residual menhaden material. Control extractions maintained a mean recovery of 87% ± 1%. GC/MS analysis time required to separate all key oil constituents outlined in Table 1 which was approximately 65 min per sample resulting in a relatively fast and simple method for extraction that provided next day results. Overall the adapted MSPD method was faster than more traditional methods such as Soxhlet extraction and more cost efficient than supercritical fluid or microwave extraction. The modified MSPD was reliable with an overall recovery of 88% ± 5%. This paper outlines a reliable and consistent method for adapting the MSPD extraction technique for the quick assessment of tissue samples during a marine pollution event.

## Figures and Tables

**Table 1:** Analytes of interest and their alkylated counterparts as identified by the Selective Ion Monitoring method developed for the identification of crude oil used to analyze menhaden tissue.

Aromatic analytes of interest		
Analyte	SIM Ion (*m*/*z*)	Retention time
Naphthalene-d_8_	**136**	**13.06**
Naphthalene	127	12.86
C1-Naphthalenes	142	16.01
C2-Naphthalenes	156	19.35
C3-Naphthalenes	170	22.14
C4-Naphthalenes	184	25.41
Acenaphthene-d_10_	**164**	**21.52**
Fluorene	166	23.37
C1-Fluorenes	180	26.17
C2-Fluorenes	194	28.81
C3-Fluorenes	208	31.04
Dibenzothiophene	184	27.19
C1-Dibenzothiophenes	198	29.31
C2-Dibenzothiophenes	212	31.33
C3-Dibenzothiophenes	226	33.54
Phenanthrene	178	27.77
C1-Phenanthrenes	192	30.56
C2-Phenanthrenes	206	32.87
C3-Phenanthrenes	220	35.10
C4-Phenanthrenes	234	37.74
Anthracene	178	27.98
Chrysene-d_12_	**240**	**41.95**
Fluoranthene	202	33.87
Pyrene	202	34.32
C1-Pyrenes	216	36.09
C2-Pyrenes	230	38.29
C3-Pyrenes	244	40.72
C4-Pyrenes	258	42.40
Naphthobenzothiophene	234	38.94
C1-Naphthobenzothiophenes	248	40.66
C2-Naphthobenzothiophenes	262	42.52
C3-Naphthobenzothiophenes	276	44.70
Benzo(a)Anthracene	228	40.09
Chrysene	228	40.24
C1-Chrysenes	242	42.09
C2-Chrysenes	256	43.88
C3-Chrysenes	270	46.16
C4-Chrysenes	284	47.68
Perylene-d_12_	**264**	**48.64**
Benzo(b)fluoranthene	252	45.25
Benzo(k)fluoranthene	252	45.30
Benzo(e)pyrene	252	45.89
Benzo(a)pyrene	252	46.07
Perylene	252	46.56
Indeno(1, 2, 3-cd)pyrene	276	51.50
Dibenzo(a, h)anthracene	278	51.23
Benzo(g, h, i)perylene	276	52.22

Bold: internal standard.

**Table 2:** Assessment of phenanthrene d_10_ recovery using a modified MSPD protocol.

Treatment	Whole fish (mean dry wt. in g)	Mean fork length (cm)	% Recovery (Mean ± Std Dev.)	Corrected mean total PAHs^a^ (ng/g)	Sample (*n*)
Spiked before freeze-drying	40.16	18.25	87% (±8%)	8415	7
Spiked after freeze-drying	36.94	15.83	89% (±3%)	6485	29
Mean/total of whole study	37.56	16.30	88% (±5%)	6860	36
controls	N/A	N/A	87% (±1%)	3501	6

^a^Corrected for surrogate recovery of phenanthrene d_10_.
